# Primary cardiac angiosarcoma with multiple pulmonary metastases: A case report

**DOI:** 10.1097/MD.0000000000042235

**Published:** 2025-04-18

**Authors:** Xing Yuan, Xian-Ya Hu, Wei-Min Li, Dan Liu

**Affiliations:** a Department of Pulmonary and Critical Care Medicine, West China Hospital, Sichuan University, Chengdu, Sichuan Province, China; b State Key Laboratory of Respiratory Health and Multimorbidity, West China Hospital, Sichuan University, Chengdu, Sichuan Province, China.

**Keywords:** albumin–paclitaxel, computed tomography-guided biopsy, multidisciplinary team, primary cardiac angiosarcoma, pulmonary metastasis

## Abstract

**Rationale:**

Primary cardiac angiosarcoma is an exceptionally rare and aggressive disease characterized by high risk of metastasis and poor prognosis. Accurate diagnosis of angiosarcoma relies on pathology. For inoperable advanced patients, biopsy is essential to confirm diagnosis and treatment strategy, but obtaining tissue samples from primary cardiac site is fraught with life-threatening hemorrhage and other procedural complications. To date, the early identification and optimal management of cardiac angiosarcoma remain formidable clinical challenges. This case report aims to evaluate the effectiveness of computed tomography-guided lung metastasis biopsy as a safer and technically feasible diagnostic alternative and to propose a multidisciplinary framework, emphasizing how coordinated care optimizes diagnostic accuracy, risk stratification, and therapeutic decision-making for this complex disease.

**Patient concerns:**

A 34-year-old male patient was admitted to our hospital because of a cystic mass in the right atrium and multiple pulmonary nodules discovered at another hospital, alongside symptoms of increasing chest tightness.

**Diagnoses:**

Immunohistochemistry and gene testing confirmed the presence of angiosarcoma with lung metastases (stage IV, with NRAS mutation).

**Interventions:**

The patient received intravenous albumin–paclitaxel (100 mg/m^2^).

**Outcomes:**

The patient was discharged after 1 cycle of albumin–paclitaxel monotherapy. One- and six-month follow-up showed that after several cycles of chemotherapy, chest tightness symptoms were improved, lung metastases significantly shrank, and heart lesions stabilized, demonstrating a good therapeutic response to albumin–paclitaxel.

**Lessons:**

The limitation of this case report is the inevitable bias of a single sample. Further studies with larger samples are needed to investigate the efficacy of chemotherapy for patients with metastatic primary cardiac angiosarcoma.

## 1. Introduction

Cardiac angiosarcoma is an exceptionally uncommon malignancy arising from endothelial cells that predominantly occurs in the right atrium and pericardium, and accounts for approximately 25% to 35% of all primary malignant cardiac tumors.^[1,2]^ Patients usually seek initial medical attention due to cardiopulmonary discomfort, manifested as palpitation, heart failure, chest pain, dyspnea, and occasionally as systemic or neurological symptoms including night sweat, fatigue, fever, syncope.^[3–6]^ The rarity of this condition and its atypical symptoms often complicate targeted detection and early diagnosis. Consequently, systemic metastasis occurs frequently up to 89%, which attributes to a dismal prognosis with the median survival time ranging from 3.8 to 17 months regardless of treatment.^[7–9]^ In this report, we presented a case of primary cardiac angiosarcoma with bilateral pulmonary metastases, and discussed the clinical decisions to obtain early accurate diagnosis and optimal treatment, highlighting the importance of multidisciplinary team consultation.

## 2. Case report

A 34-year-old man presented with unexplained chest distress in August 2023, with pleural and pericardial effusion discovered after examination. Considering the possibility of tuberculosis, He was treated with HRZE (isoniazid, rifampicin, pyrazinamide, and ethambutol) quadruple antituberculosis therapy in outside hospital. Reexamination of chest computed tomography (CT) revealed a diffuse distribution of solid, partially ground-glass nodules, along with spot shadows in both lungs. Seven months later, the patients progressed with symptoms of cough, phlegm, hemoptysis, night sweat, and occasional dull chest pain. Pericardial ultrasound indicated that right atrial wall was thickened, and possibly perforated to cause pericardial hemorrhage and emboli formation inside. Positron emission tomography (PET)–CT showed a mass image in the right atrial area, measuring approximately 10.44 cm × 8.47 cm × 8.98 cm with a standard uptake value of 11.44, suggesting a malignant tumor.

**Figure 1.**
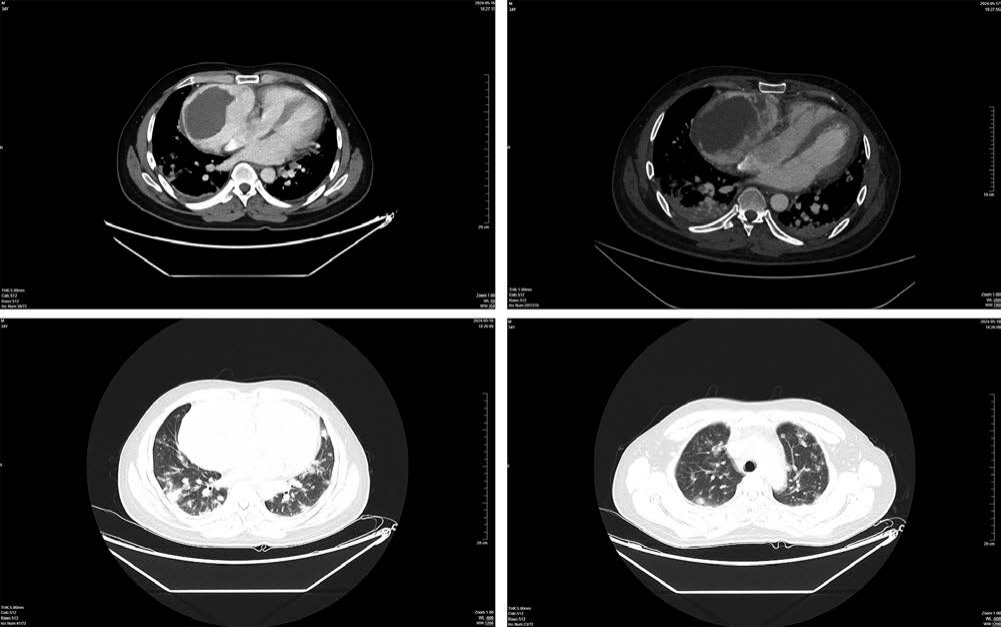
Chest computed tomography (CT) scan.

**Figure 2.**
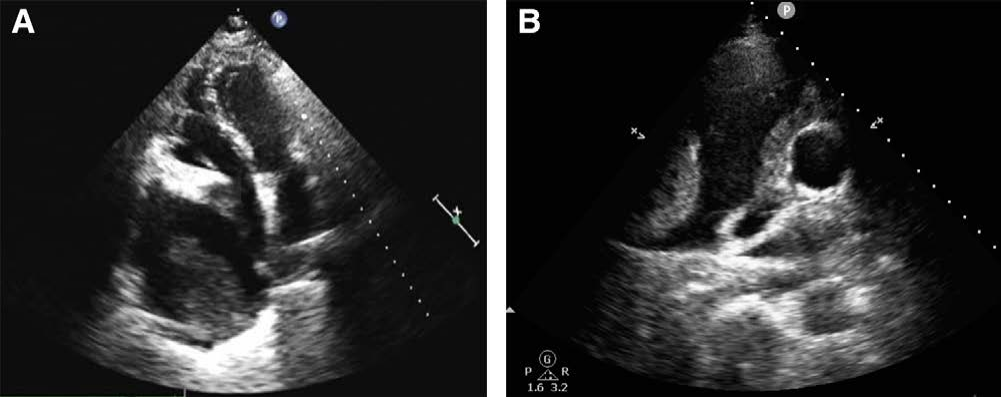
Echocardiogram. (A) Large amount of fluid accumulation in the right pericardium with solid echo mass inside, the size of which was about 54 * 71 mm, no obvious motion was observed. The right chamber was compressed and significantly deformed. (B) Irregular solid mass at the aortic root with sparse blood flow signals.

**Figure 3.**
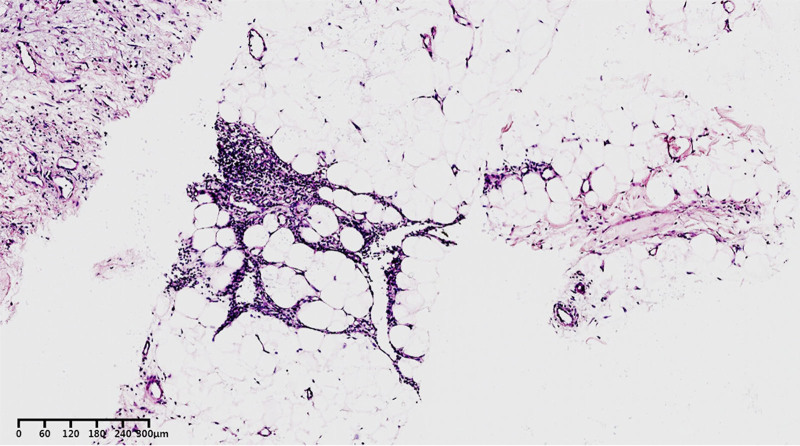
Pathological section of mediastinal biopsy demonstrating atypical tumor cells.

**Figure 4.**
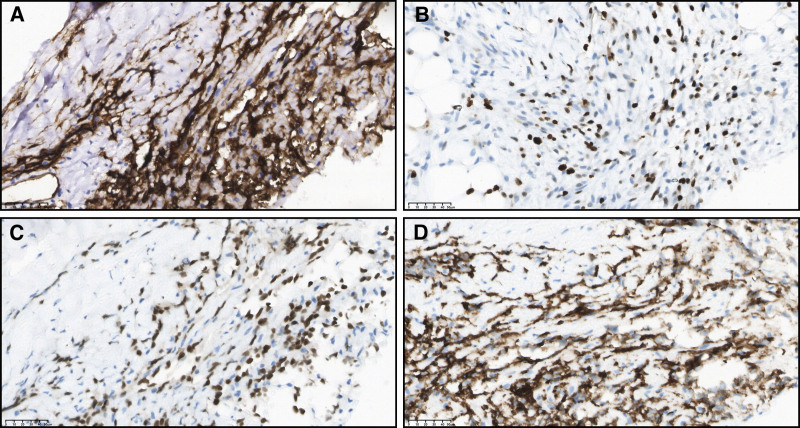
Immunohistochemistry results. (A–D) CD34 (+), Ki67 (approximately 20%), ERG (+), CD31 (+).

Nine months after the onset of symptoms, he was admitted to our hospital. Physical examination showed that the heart boundary was enlarged, and grade III/6 systolic murmurs could be heard at the 5th intercostal border of the left sternum. Contrast-enhanced CT scan displayed multiple pulmonary nodules and neoplastic lesions with thrombosis in the right atrium (Fig. 1), consistent with previous hospital. Repeated echocardiography presented cystic structure in the right edge of the heart and an irregular solid mass around the aortic root compressing the right atrium (Fig. 2). In order to discuss the following management, we convened a multidisciplinary team (MDT) discussion involving specialists in cardiology, cardiac surgery, thoracic surgery, and oncology. Based on the medical history and imaging evidence, a malignant lesion with extensive lung metastases was suspected. Due to the extremely thin atrial wall and the high risk of cardiac rupture, the feasibility of a myocardial biopsy or surgery was deemed low. Hence, we conducted CT-guided percutaneous lung and mediastinal biopsies repeatedly to determine the nature of the lesion. Pathological specimens of mediastinal puncture revealed a few atypia cells (<50) adjacent to adipose tissue (Fig. 3). The immunohistochemistry results were as follows (Fig. 4): CD34 (+), Ki67 (+, approximately 20%), PCK (‐), CK5/6 (‐), TTF-1 (‐), p63 (‐), desmin (‐), TdT (‐), mpo (‐), LCA (‐), SALL4 (‐), HHV8 (‐). The preliminary pathological findings suggested hematogenous tumors. Before the further results of the tissue sample examination had been obtained, the patient’s condition deteriorated 20 days after admission with symptoms of intermittent hemoptysis, exercise-induced dyspnea, paroxysmal dizziness and other compressive symptoms. MDT consultation reconvened, and after combining the opinions of the Department of cardiology, oncology, thoracic surgery and vascular surgery, we concluded that there were no indications for surgery because of tumor invasion of the heart and large blood vessels. Based on the pathological diagnosis of vascular tumors, we proposed a combined immunotherapy of gemcitabine and albumin–paclitaxel. Further, it was still necessary to clarify the immunohistochemistry of CD31 and ERG, as well as to clarify the target by solid tumor 1021 gene detection before we formulated the final chemotherapy plan. Supplementary immunohistochemistry results (Fig. 4) showed ERG (+), CD34 (+), CD31 (+), SALL4 (‐), OCT3/4 (‐), TFE-3 (‐), Ki67 (+, approximately 10%–20%). Additionally, comprehensive targeted gene testing of the solid tumor demonstrated type II mutation of NRAS c.38G>T (p.G13V) EX 2 with a mutation abundance of 3.9%.

Ultimately, the patient was diagnosed with angiosarcoma derived from endothelial cells of vascular origin with multiple lung metastases (stage IV, with NRAS mutation). And the patient received chemotherapy with intravenous albumin–paclitaxel (100 mg/m^2^). After 1 cycle of this chemotherapy, the patient was discharged in stable condition after 44 days of hospitalization. One month after discharge, the patient was revisited with second cycle of chemotherapy performed in external hospital. And the patient reported improved chest tightness, and subsequent CT scans showed significant reduction of the pulmonary nodules with stable heart lesions. In order to seek medical preparation for subsequent treatment, the patient completed the 8th cycle of albumin–paclitaxel monotherapy in outside hospital and was readmitted 6 months after discharge. Blood routine and liver and kidney function tests were completed, and it was found that leukocyte and neutrophil decreased to II degree, which was considered as bone marrow suppression after chemotherapy. Echocardiogram showed local pericardial effusion with solid echo mass, which was reduced compared with the last examination. After completing chest and abdomen CT and brain magnetic resonance imaging, the efficacy was evaluated as partial response. After fully communicating with the patient about his condition and systemic treatment plan, the patient requested the treatment of albumin–paclitaxel plus toripalimab. Because the treatment time of the next cycle was not reached, the patient requested the next cycle of treatment outside the hospital and was discharged.

## 3. Discussion and conclusion

This case report documents primary cardiac angiosarcoma with multiple pulmonary metastases. The rare malignancy is characterized by its infiltrated nature and nonspecific symptoms, which complicate the diagnostic process. Our patient began with chest tightness and appeared cough, phlegm, hemoptysis, night sweats during the course of the disease. The outside hospital gave antituberculosis treatment and levofloxacin anti-infection treatment according to the symptoms, but the effect was poor, and the patient`s condition worsened 7 months later. Imaging plays a crucial role in early detection and initial evaluation of space-occupying lesions. Echocardiography shows high sensitivity and specificity over 90% in assessing cardiac masses, making it an essential first-line tool.^[10]^ Other imaging modalities, like X-rays, CT scans, and magnetic resonance imaging, provide additional diagnostic support. In addition, PET–CT offers metabolic information, particularly useful for assessing metastasis and treatment response.^[11]^ Moreover, cytologic examination of pericardial fluid also helps in some condition. Nevertheless, the definitive diagnosis cannot be determined solely based on imaging and normal laboratory tests. Histopathological examination remains the golden standard to confirm the nature of cardiac masses, and immunochemistry reveals distinct endothelial markers associated with angiosarcoma.^[12]^ Specifically, CD31, CD34, ERG, and FVIII are typical markers to confirm the endothelial origin of the tumor.^[4,13]^ In this case, the patient’s heart occupying was quickly identified as right atrial neoplastic lesions with thrombosis at the initial stage of admission based on PET–CT of other hospital, CT scan and three-dimensional vascular enhanced scan after admission, as well as abnormal elevation of tumor makers. Whereas, whether the pulmonary space-occupying lesions were the result of infection or malignant metastasis was still unclear. Therefore, we examined tuberculosis indicators, cryptococcus antigen, GM test, fungal G test to rule out tuberculosis and fungal infections. Furthermore, we continued to test the drained pleural fluid. However, the pleural fluid smear and culture were all negative, and cytology examinations revealed no presence of tumor cells. And the final diagnosis was confirmed by histopathology. To obtain specimen in a less invasive way, transthoracic puncture biopsy and transvenous endomyocardial biopsy are frequently used. Given to the high risk of cardiac rupture, we performed CT-guided percutaneous lung biopsy to support the diagnosis of primary cardiac angiosarcoma with secondary metastasis. Notably, after the first 2 pathological examinations, we failed to evaluate the immunohistochemistry of CD31 and ERG because of insufficient specimen. Hence, in the selection of biopsy strategies for patients with angiosarcoma, safety and small sample false negative issues need to be considered in order to choose appropriate biopsy sites and avoid the repeated biopsies.

Throughout the whole process, MDT greatly promoted risk warning, differential diagnosis, and treatment planning. In terms of risk warning, we were consciously vigilant to the risk of pericardial tamponade, heart rupture, and pulmonary infarction due to the right atrial cancer embolus and thrombus. According to the advice of cardiology and oncology, we avoided the high-risk pericardial puncture and myocardial biopsy, and performed puncture biopsy from the lung lesions. In addition, β-HCG and AFP tests were supplemented to rule out tumors of gonadal origin with similar imaging features of this patient. For the treatment of cardiac angiosarcoma, early surgical resection, when feasible, is the first option, and multimodality protocol combined with chemo-radiotherapy is also recommended by experts with a median progress-free-survival for about 15 months.^[14,15]^ Beyond that, heart transplantation can be used as an alternative to macroscopical complete resection for patients with tumor can’t be removed through conventional surgery and without metastasis.^[16]^ For patients with malignant metastasis like this case, surgical benefits are limited, and chemotherapy or targeted therapy may be a better option to improve the chances of survival.^[17]^ Doxorubicin plus ifosfamide and gemcitabine plus docetaxel are common chemotherapy combination to treat sarcomas.^[18]^ And we also noticed the application of paclitaxel plus doxorubicin and albumin-bound paclitaxel plus immunotherapy like tislelizumab.^[8,19]^ In the treatment of this patient, considering the vascular origin of this tumor, gemcitabine and albumin–paclitaxel combined with immunotherapy was our preliminary option, which was consistent with previous studies. After the pathologic results were confirmed, owing to the poor basic condition of the patient, we gave albumin–paclitaxel monotherapy, and the alternative was lipid doxorubicin, the addition of bevacizumab depended on the disease progression. To find further targets, we performed solid tumor genetic testing that revealed mutations in the NRAS, FLT4, and KDR genes. Although there are recently no targeted drugs for these mutated genes, this is a promising method to pursue targeted therapy. After 1 cycle of intravenous albumin–paclitaxel chemotherapy, the patient was discharged, and the follow-up showed improvement in the metastatic lesions, possibly resulting from a relatively low Ki67 index compared to previous cases.

Currently, there is no standard therapy for cardiac angiosarcoma, and the role of adjuvant chemotherapy and neoadjuvant systemic therapy is still controversial.^[20]^ As a case report, it can provide some ideas for the diagnosis and treatment of cardiac angiosarcoma, but inevitable differences exist, calling for randomized clinical trials and further investigation on large sample in this area to promote early diagnosis and treatment guidance.

## Author contributions

**Conceptualization:** Dan Liu.

**Resources:** Xing Yuan.

**Visualization:** Xian-Ya Hu.

**Writing – original draft:** Xian-Ya Hu.

**Writing – review & editing:** Xing Yuan, Wei-Min Li, Dan Liu.
